# Childhood Rabies Deaths and the Rule of Rescue

**DOI:** 10.3390/tropicalmed2020009

**Published:** 2017-04-17

**Authors:** David Durrheim

**Affiliations:** 1 School of Medicine and Public Health, University of Newcastle, Callaghan, New South Wales 2387, Australia; David.Durrheim@newcastle.edu.au; Tel.: +61-2-49246395; 2 College of Public Health, Medical and Veterinary Sciences, James Cook University, Queensland 4814, Australia

**Keywords:** rabies, child, death, vaccination, immunization, ethics, rights, beneficence, rescue

## Abstract

Every childhood rabies death is potentially preventable. The vaccine that prevents rabies disease has a formidable safety and efficacy track record. Rabies vaccination of dogs and timely pre-and post-exposure vaccine administration are life-saving and cost-effective, and yet nearly 60,000 people, mainly children, die unnecessarily each year. Poor performance by many veterinary and public health systems, and neglect by complicit authorities is in stark contravention of the Convention on the Rights of the Child. The ethical principle of beneficence and the rule of rescue demand re-energised commitment to eradicating childhood rabies deaths.

## 1. Introduction

The burden of human rabies falls heavily on young children in developing countries with most of the approximately 59,000 annual deaths due to rabies occurring in children younger than 15 years of age in Africa and Asia with virus transmission largely from rabid dogs [1,2]. Almost every one of these deaths reflects a failure of the public health and veterinary systems as the vaccine that is available is highly effective in preventing disease in dogs and humans [3]. However, in addition to the primary failure of not reaching all dogs with vaccine by weak veterinary programs, and the secondary failure in not providing ready access to post-exposure treatment with rabies vaccine and immunoglobulin by deficient health systems, either through inability to secure stock or to follow guidelines, there is a potential third failure, that of not administering pre-emptive protective vaccination to children in high-risk rabies-endemic areas where the perpetual weakness of the public health and veterinary systems in delivering education and vaccination is well recognised [4]. A recent systematic review of the safety and immunogenicity of pre-exposure rabies prophylaxis found that it is safe, effective and should be considered in areas “where access to post-exposure prophylaxis is limited or delayed, where the risk of exposure is high and may go unrecognised, and where controlling rabies in the animal reservoir is difficult” [5]. However, only two countries, Peru and the Philippines, have thus far implemented this strategy.

In addition, accelerated regimens (all vaccine doses administered within a week) and administration by intradermal rather than intramuscular route were found to be highly immunoprotective. Thus we have at our disposal effective tools to eliminate childhood rabies deaths but to date the will to fully embrace this challenge in many endemic countries and the international community has been lacking.

## 2. Convention of the Rights of the Child

It is timely to remind ourselves of our shared obligations under the Convention of the Rights of the Child (http://www.unicef.org/crc/). This international legally binding instrument enjoys remarkable acceptance with all United Nations states, excepting Somalia and the United States of America, having ratified the Convention [6]. Article 6 (Survival and Development) is particularly pertinent to the childhood rabies death sentence: “Children have the right to live. Governments should ensure that children survive and develop healthily”. Thus individual governments in rabies endemic areas should not tolerate poor performance of their veterinary or public health sectors in achieving high dog rabies vaccination or ensuring the availability of post-exposure rabies vaccination, respectively. Each rabies death should prompt an enquiry to identify preventable system failures. Confidential enquiries into maternal deaths, infant deaths, peri-operative deaths, and malaria and cholera deaths have proven immensely valuable for correcting system weaknesses. Identifying the system root causes of deaths would allow targeting of resources to limit the likelihood of recurrence. Governments may argue that this will come at an opportunity cost as there are many competing priorities for finances. Sustainable development requires investment in many areas including clean water, adequate sanitation, quality education and secure food supply. However, immunisation is a wonderful public good, in that a single intervention can provide long-lived benefits to the individual. Further, dog rabies vaccination programs are a basic indicator of the coverage and quality of veterinary public health initiatives. Recent case studies from Bhutan and Tanzania demonstrated the effectiveness of programs implemented to achieve at least 70% canine coverage [7].

A health service that does not effectively reach communities with education messages about stray canine and wild life avoidance, and appropriate first aid measures, or cannot provide a reliable timely supply of potent rabies vaccine, will likely be failing to provide other primary health care services. Both dog rabies vaccination and human pre-exposure (in certain high risk settings) and post-exposure rabies vaccination have been shown to be cost-effective in developing countries, and for canine rabies vaccination, cost-saving (if the estimated $2.7 billion wasted with post-exposure prophylaxis annually is included), so it is time for individual governments to demonstrate appropriate accountability and deliver on their human rights commitment [5,7].

A true commitment to the rights of children demands that every country’s government implements carefully monitored strategies for guaranteeing all children protection against rabies through equal access to effective vaccines based on local rabies epidemiology [8].

But impoverished endemic countries should not shoulder this responsibility alone. Article 24 (Health and Health Services) states: “Children have the right to good quality health care—the best health care possible—to safe drinking water, nutritious food, a clean and safe environment, and information to help them stay healthy. Rich countries should help poorer countries achieve this”. This places a specific legal obligation on developed countries to support less-developed countries to ensure social justice in the delivery of preventive and curative health care, including rabies prevention. These developed countries have already experienced the benefit of eradication of canine rabies through well organized and funded veterinary vaccination campaigns. GAVI, the global alliance for vaccines and immunization, has provided a dependable and accountable mechanism for raising and administering funds from donors and wealthy countries for critical vaccine introductions and immunisation program strengthening in developing countries. It is time that GAVI carefully reviewed the evidence supporting expanding access to rabies vaccine and immunoglobulin, including the childhood mortality burden, the cost-effectiveness data and the opportunity for strengthening immunisation programs.

## 3. Principle of Beneficence

This legally binding prerogative for eliminating childhood rabies deaths is supported by important ethical considerations. The principle of beneficence is succinctly summarised by the Golden Rule: “Do unto others, as you would have them do unto you”. This ethical principle dictates that national governments and the international community have a duty of care to ensure that all children enjoy the protection offered by effective vaccines [9]. The strength of the duty of care depends on the availability of effective and affordable measures [10]. This requirement is clearly satisfied by rabies vaccines, which enjoy a proven track record if administered correctly without delay.

## 4. Rule of Rescue

The rule of rescue places a compelling obligation on those that are able, in this case governments and health personnel, to “rescue identifiable individuals facing avoidable death” if personal sacrifice is not excessive [11]. This duty is influenced by the urgency of the situation, the consequences of doing nothing, the feasibility of preventing serious consequences and the sacrifice required [12]. Rabies vaccination for all children who are at high risk of exposure or have been exposed to a bite or scratch from an infected animal easily meets each one of these criteria: “urgency”—delayed vaccination can result in preventable death; “consequence of doing nothing”—death is almost inevitable once clinical symptoms have occurred [2]; “feasibility of preventing serious consequences”—vaccine and immunoglobulin are highly effective and cost-effective in preventing disease; and “sacrifice required”—surely the opportunity costs of providing vaccine in these circumstances are morally defensible! [13]

## 5. Conclusions

The principle of Justice obligates those who are better off to assist those who are worse off and to allocate resources accordingly [14]. A global Convention considers the lives of children as precious and demands that governments ensure child health and survival. We have effective tools to rescue children from agonising preventable deaths due to lyssavirus 1. Rabies deaths in children are a true measure of our generation’s commitment to children’s rights and social justice.
